# Proton-Detected Solid-State NMR Spectroscopy of Bone with Ultrafast Magic Angle Spinning

**DOI:** 10.1038/srep11991

**Published:** 2015-07-08

**Authors:** Kamal H. Mroue, Yusuke Nishiyama, Manoj Kumar Pandey, Bo Gong, Erin McNerny, David H. Kohn, Michael D. Morris, Ayyalusamy Ramamoorthy

**Affiliations:** 1 Department of Biophysics, University of Michigan, Ann Arbor, Michigan, 48109-1055, United States; 2 Department of Chemistry, University of Michigan, Ann Arbor, Michigan, 48109-1055, United States; 3 JEOL RESONANCE Inc., Musashino, Akishima, Tokyo 196-8558, Japan; 4 RIKEN CLST-JEOL Collaboration Center, Tsurumi, Yokohama, Kanagawa 230-0045, Japan; 5 School of Dentistry, University of Michigan, Ann Arbor, Michigan, 48109-1078, United States

## Abstract

While obtaining high-resolution structural details from bone is highly important to better understand its mechanical strength and the effects of aging and disease on bone ultrastructure, it has been a major challenge to do so with existing biophysical techniques. Though solid-state NMR spectroscopy has the potential to reveal the structural details of bone, it suffers from poor spectral resolution and sensitivity. Nonetheless, recent developments in magic angle spinning (MAS) NMR technology have made it possible to spin solid samples up to 110 kHz frequency. With such remarkable capabilities, ^1^H-detected NMR experiments that have traditionally been challenging on rigid solids can now be implemented. Here, we report the first application of multidimensional ^1^H-detected NMR measurements on bone under ultrafast MAS conditions to provide atomistic-level elucidation of the complex heterogeneous structure of bone. Our investigations demonstrate that two-dimensional ^1^H/^1^H chemical shift correlation spectra for bone are obtainable using fp-RFDR (finite-pulse radio-frequency-driven dipolar recoupling) pulse sequence under ultrafast MAS. Our results infer that water exhibits distinct ^1^H−^1^H dipolar coupling networks with the backbone and side-chain regions in collagen. These results show the promising potential of proton-detected ultrafast MAS NMR for monitoring structural and dynamic changes caused by mechanical loading and disease in bone.

Over the past years, there has been growing interest in obtaining molecular-level structural and dynamic information on the various constituents of bone. Such information could provide relevant insights into bone biomechanical function and the effects of bone diseases and aging. The complex nature of bone, however, poses tremendous challenges to the application of traditional biophysical techniques. Fortunately, recent studies have demonstrated that non-invasive solid-state NMR experiments are capable of providing valuable insights into the structure of bone^1^,^2^,^3^,^4^,^5^,^6^. However, the low-resolution spectral lines and long measurement times have considerably limited the effective utilization of high-resolution solid-state NMR techniques^7^,^8^. In this study, we demonstrate a high-throughput solid-state NMR approach for studies on bone.

Recent advances in solid-state magic angle spinning (MAS) NMR spectroscopy have provided excellent opportunities to obtain key high-resolution information on the structure and dynamics of complex biomolecules that are amenable neither to solution-state NMR studies nor to microscopic and diffraction analyses because of their large size, poor solubility, and/or inherent non-crystalline nature^9^,^10^,^11^,^12^,^13^. Particularly, tremendous progress has been made over the last several years in the design of NMR probes, so that spinning frequencies up to 100–110 kHz can now be achieved using 0.75-mm outer-diameter MAS rotors^14^,^15^,^16^,^17^,^18^. One of the advantages of these ultrafast MAS capabilities, especially in proton-based experiments, is the remarkable improvement in spectral resolution owing to the effective suppression of strong ^1^H–^1^H dipolar couplings that cause significant line broadening in ^1^H NMR spectra of solids^19^,^20^. Another advantage pertaining to ultrafast spinning is the increase in the transverse relaxation time *T*_2_, which also leads to resolution and sensitivity enhancement, and enables the manipulation of long-lived magnetization for potential applications^18^,^21^,^22^. In addition, the extremely small size of the ultrafast MAS rotors enables the use of much smaller sample quantity (e.g., a 0.75-mm rotor accommodates ~300 nL sample)^17^,^23^, which is particularly beneficial for samples that can only be available in sub-milligram amounts; the limited sample volume that can fill such ultrafast MAS rotors is compensated, to a large degree, by a high filling factor of the sample radio-frequency (RF) coils. Moreover, measurements under ultrafast MAS conditions benefit from the use of low or no proton decoupling power to avoid sample overheating in homo/hetero-nuclear experiments (see Ref. 24 and references therein). Ultrafast MAS NMR spectroscopy with proton detection thus represents a promising and attractive approach for the structural studies of macromolecules, and its strong potential to provide atomic-level insights into such systems has been recently demonstrated in several instances^16^,^17^,^18^,^25^,^26^,^27^.

While ultrafast MAS NMR has been used to study the structure in microcrystalline proteins and fibrils as well as small organic solids, the applicability of this approach to characterize complex heterogeneous biomaterials like bone has not yet been explored. Bone, one of the highly heterogeneous and challenging biological materials, displays a complex structural hierarchy. At the nanoscopic level, a protein matrix consists of tropocollagen molecules, each built from three type I collagen polypeptide strands twisted together into a triple helix. The helices align themselves to form three-dimensional fibrils around and within which nanoparticles of poorly crystalline carbonated hydroxyapatite mineral are embedded. This complicated assembly of collagen fibrils and mineral nanocrystals is supported by water and other non-collagenous macromolecules (*i.e.,* proteins, lipids, polysaccharides, and citrates that account for ~10% of the bone organic phase)^28^,^29^,^30^.

Using a 0.75-mm NMR probe, we demonstrate the impact of ultrafast MAS to provide the necessary spectral resolution and sensitivity for obtaining useful solid-state ^1^H NMR spectra of bone. We also demonstrate the use of 2D ^1^H/^1^H fp-RFDR (finite-pulse radio frequency-driven dipolar recoupling)^31^,^32^ based chemical shift correlation experiments under 100 kHz MAS to investigate the atomic-level interactions that exist among the various constituents of bone, and specifically to further understand the role played by water in the stabilization and self-assembly of collagen triple-helical conformation as well as its impact on the nucleation and distribution of the bone mineral nanoparticles within the organic matrix. It is well known that due to poor spectral resolution caused by the combined effect of strong ^1^H–^1^H homonuclear dipolar couplings and narrow ^1^H chemical shift range, 2D ^1^H/^1^H MAS experiments on solids have been a long-standing challenge. However, the ability to achieve ultrafast MAS rates (60 kHz and higher) facilitates the collection of useful ^1^H-detected solid-state NMR spectra, and hence provides a timely opportunity for the employment of these ^1^H-based fp-RFDR experiments on rigid solids, particularly those characterized by their structural heterogeneity and complexity, such as bone tissues. Despite that very fast MAS frequencies can cause a reduction in the spin diffusion rate among protons in a solid framework, the recoupling of the ^1^H–^1^H dipolar interactions by a rotor-synchronized π-pulse train during the mixing time of the fp-RFDR pulse sequence renders the ^1^H–^1^H spin diffusion processes rapid and effective even in the ultrafast MAS regime^14^,^31^,^32^,^33^,^34^. In this context, recent studies have demonstrated the feasibility of these experiments under ultrafast MAS on small organic solids as well as under slow MAS on model membrane-bound peptides and proteins^15^,^23^,^25^,^26^,^27^,^35^,^36^,^37^,^38^,^39^, which are typically mobile solids with relatively weak proton-proton dipolar couplings.

## Results and Discussion

**Figure 1:**
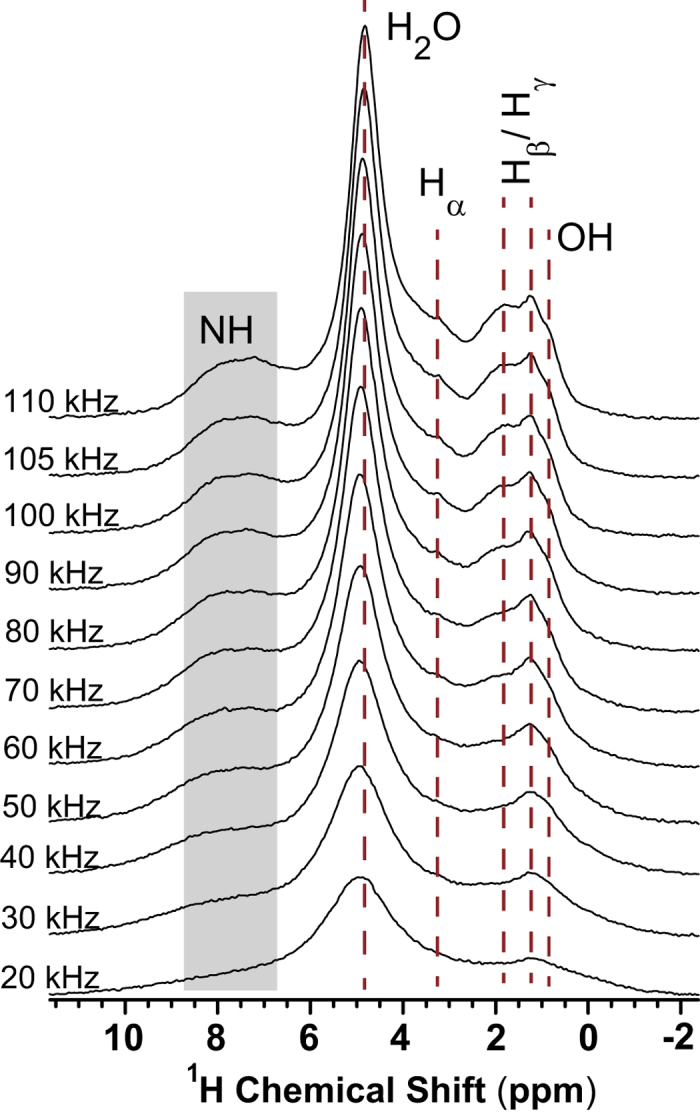
^1^H MAS NMR spectra recorded at 14.1 T from bovine cortical bone under multiple MAS rates. The spectra were acquired by co-adding 4 transients with a rotor-synchronized spin- echo pulse sequence using a 0.75-mm MAS HX probe with a 0.72 μs ^1^H 90° pulse and a 3 s recycle delay.

In this study, we systematically explore the benefits of employing ultrafast MAS frequencies in ^1^H-detected one- and two-dimensional NMR experiments for the purpose of enhancing the resolution and sensitivity of ^1^H MAS NMR spectra for bone. Experimental results obtained from powder bovine cortical bone samples are presented and interpreted, taking into account the complex heterogeneous structure and composition of bone, which result in complications arising from overlapping ^1^H NMR signals of the various organic and mineral constituents of bone. The ^1^H MAS spectra of bone recorded at multiple MAS frequencies up to 110 kHz MAS using a rotor-synchronized spin-echo sequence on a 600-MHz NMR spectrometer are presented in Fig. 1. Overall, we observe that increasing the MAS frequency produces a progressively better resolved and more sensitive spectral line shapes. At moderate-to-fast MAS rates (20–50 kHz), the spectrum exhibits only two peaks, with the water peak at ~5 ppm dominating the spectra. It is believed that water, the third major component in bone, plays a pivotal role in its biomechanical behavior, including stabilizing the matrix and contributing to its ductility. Water accounts for about 10% of fresh bone weight and occupies ~15–25% of its volume^40^. Nevertheless, studies have shown that the amount of water in bone decreases with age and with skeletal growth^41^,^42^. Bone water is found in different forms with various binding conditions: free (or mobile) water filling the bulk of the microscopic pores in the calcified matrix, water bound to organic matrix mainly in the collagen network and organic-mineral interface, and water associated with the mineral phase^43^,^44^,^45^. In addition to the strong water signal, the other broad peak centered at ~1.2 ppm originates from multiple overlapping signals that correspond to aliphatic side-chain protons in the bone organic matrix as well as to the structural OH groups in bone mineral. The line-narrowing advantage of MAS is clearly manifested in these spectra upon increasing the spinning frequency, where better resolution is obtained in the ultrafast MAS regime (ν_R_ ≥ 60 kHz). Here, a gradual resolution of the aliphatic region (0.5–3 ppm) is observed, where peaks from protons in the side chains of the amino acid residues in the organic matrix can now be observed. Notably, the broad peak centered at ~7.5 ppm in the (6–8 ppm) region, which comes from amide NH protons associated with the peptide bonds in collagen and which is almost invisible at lower MAS rates, becomes narrow and clearly more visible as the MAS frequency reaches its highest limits. Similarly, the peak at ~3.5 ppm, which is most likely due to α-protons of glycine (the most abundant amino acid residue in collagen), can only be resolved at ultrafast MAS rates. At 100 kHz MAS and beyond, the spectrum displays fairly resolved peaks for the structural hydroxyl group from the bone mineral, and for the side-chain protons from the organic matrix, as well as for the amide NH groups.

**Figure 2:**
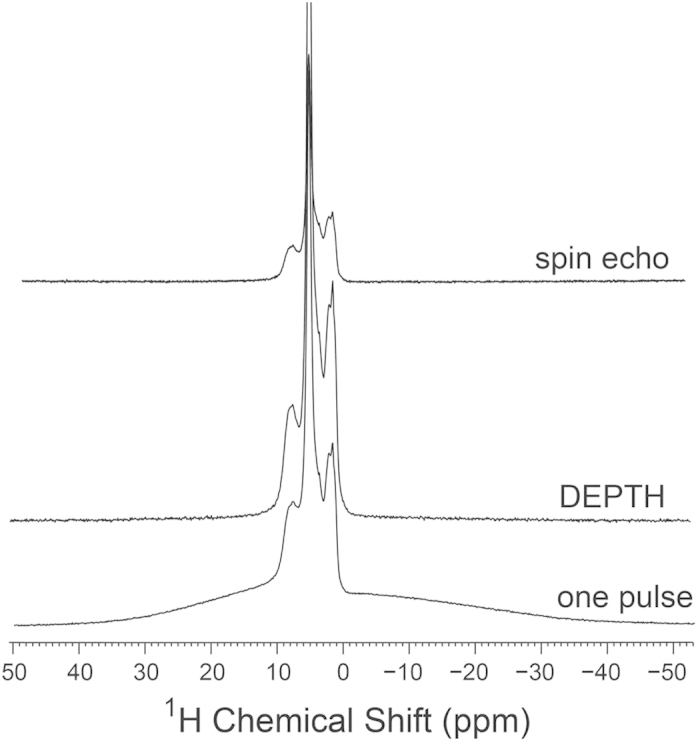
^1^H MAS NMR spectra of bovine cortical bone at 14.1 T (600.17 MHz ^1^H Larmor frequency) with 110 kHz MAS frequency. The spectra were acquired with either a ^1^H one-pulse excitation (bottom), a DEPTH pulse sequence (middle), or a rotor-synchronized spin-echo (top), using a 0.75-mm MAS HX probe with a 0.72 μs π/2 ^1^H-pulse, a 3 s recycle delay. 16 transients were collected for each of the one-pulse and DEPTH spectra while the spin-echo spectrum was acquired in 4 transients.

A common issue regularly encountered in ^1^H MAS NMR experiments on solids is the appearance of a broad unwanted signal in the spectrum. This signal, commonly called the background signal, originates from protons found in locations other than the sample under study, usually in the materials used to build the NMR probehead (or the stator), MAS rotor or other accessories used inside the probe. The background signal can be significantly problematic if the signal arising from the sample is inadequately weak or if it is too broad, in which case it is difficult to distinguish the sample signal from that of the background. To investigate the presence of background signals and their effects on NMR spectra of bone samples under the ultrafast MAS rates used here, we recorded a conventional single-pulse (Bloch decay) spectrum and another spectrum using the DEPTH pulse sequence at 110 kHz MAS, and compared them with the rotor-synchronized spin-echo spectrum of bone, as shown in Fig. 2. The DEPTH pulse sequence is a common method used for effective ^1^H background signal suppression, and it consists of a π/2 pulse followed by two π pulses that are phase cycled according to a combined “EXORCYCLE” and “CYCLOPS” scheme^46^,^47^,^48^. Hence, only ^1^H spins located at the center of the radiofrequency coil will be excited and consequently contribute to the ^1^H MAS spectrum, while those outside the RF coil center will encounter a low RF field and are therefore not detected. Despite the relatively high abundance of ^1^H nuclei in bone, the one-pulse spectrum in Fig. 2 suffers from a strong background signal that appears as a distinctive broad signal centered at ~5 ppm, thus causing a poor baseline. The background signal, however, is efficiently suppressed with the DEPTH sequence and the baseline is largely improved. Moreover, although the DEPTH pulse experiment for ^1^H MAS NMR usually results in a considerable loss of sample signal intensity in comparison to the conventional one-pulse experiment, our results reveal that the sample signal is largely retained in the DEPTH spectrum. This may be attributed to the strong ^1^H RF field strength applied (*ca.* 350 kHz) in the DEPTH experiment and the small size of the RF coil of the 0.75-mm probehead used for acquiring these spectra, which means that the whole sample is located in the center of the coil. Of note is that the same number of scans was used to collect both spectra. In a manner similar to the DEPTH method, the spin-echo spectrum of bone, which is based on the transverse relaxation of excited resonances, also shows an effective suppression of the background signal owing to its faster transverse relaxation rate relative to other ^1^H resonances in the spectrum.

**Figure 3:**
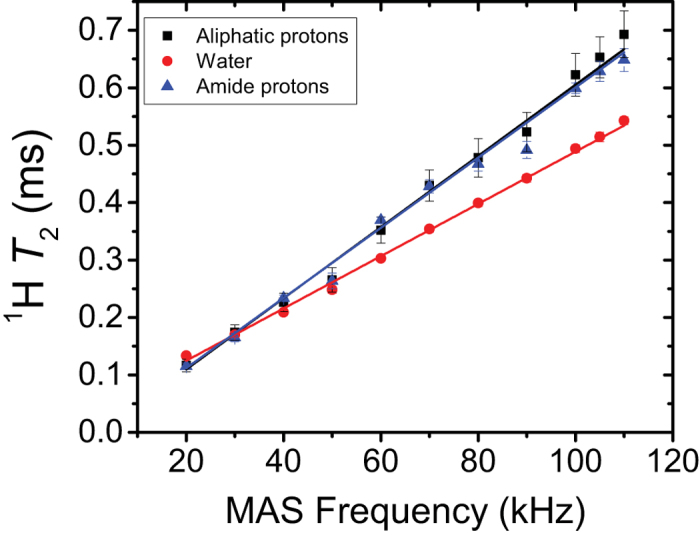
Average ^1^H spin-spin relaxation time (*T*_2_) of powdered cortical bone as a function of MAS frequency. The ^1^H *T*_2_ values were determined by ^1^H-detected spin-echo NMR experiments with an incremented echo delay at 14.1 T. The reported errors were estimated from the best-fitting of experimental data.

To explore the advantages of using faster MAS rates for efficient proton detection and their impact on spectral resolution and sensitivity, the ^1^H *T*_2_ spin-spin (transverse) relaxation times were measured using the spin-echo sequence with an incremented echo delay for each of the three main bulk regions in the ^1^H NMR spectra of bone at multiple spinning frequencies that ranged from 20 to 110 kHz. These *T*_2_ time constants were obtained by fitting the measured peak integral intensities to a mono-exponential decay function, and are plotted in Fig. 3 as a function of the MAS rotor frequency. Overall, the measured *T*_2_ times were found to show linear increase with the spinning frequency, which implies that the observed average proton linewidths decrease linearly with the spinning frequency. Even at the highest spinning frequencies (100 kHz and higher) used here, the ^1^H *T*_2_ is still found to increase directly in relation to the MAS frequency. Between 70 kHz and 110 kHz MAS frequency, for example, the observed average *T*_2_ of the amide protons was found to increase from 0.43 ms to 0.65 ms, confirming the linear decrease in proton linewidth with rotor frequency. This indicates that the line broadenings in the measured ^1^H NMR spectra of bones are mainly homogeneous and are due to a strong network of ^1^H–^1^H dipolar couplings, and hence would be narrowed further by spinning at even higher than 110 kHz MAS frequency.

**Figure 4:**
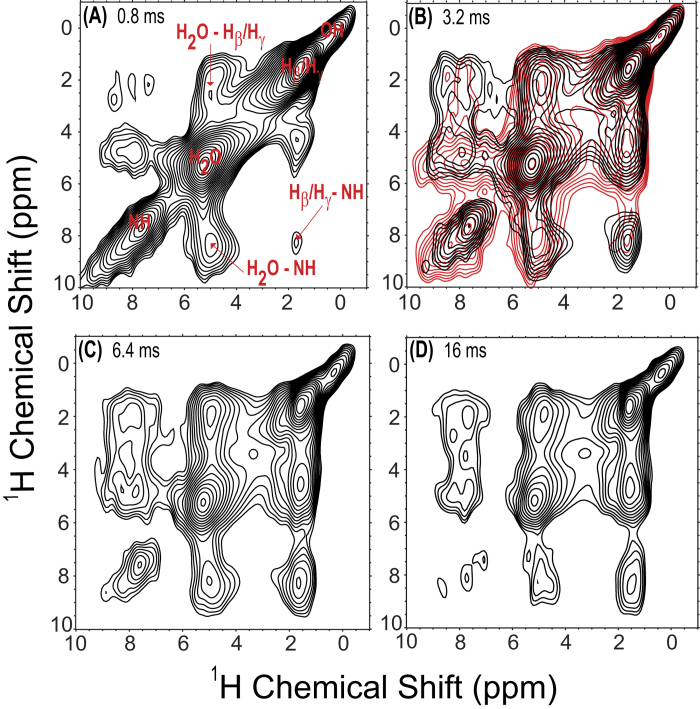
Representative 2D ^1^H/^1^H chemical shift correlation NMR spectra of bone at 14.1 T under 100 kHz MAS with fp-RFDR mixing times of (**A**) 0.8 ms, (**B**) 3.2 ms, (**C**) 6.4 ms, and (**D**) 16 ms. Shown also in (**B**) is a 2D NOESY spectrum (in red) with a 32 ms mixing time overlaid on the 3.2-ms mixing time RFDR spectrum.

In order to obtain further insights into ^1^H-detected MAS NMR spectra of the bone samples at the highest possible resolution, we further investigated the effect of ultrafast MAS on 2D ^1^H/^1^H fp-RFDR based chemical shift correlation experiments. Figure 4 shows the 2D ^1^H/^1^H spectra of bone acquired under 100 kHz MAS with different mixing times, along with one 2D ^1^H/^1^H NOESY (Nuclear Overhauser Effect SpectroscopY) spectrum superimposed on one of them (Fig. 4B) for comparison. The sole difference between the RFDR and NOESY experiments is that while a train of rotor-synchronized π pulses is introduced in the mixing time of the RFDR experiment, no such pulses are applied in the NOESY experiment. This train of π pulses in the RFDR sequence serves to recover the zero-quantum (known as flip-flop) part of the ^1^H-^1^H homonuclear dipolar coupling term of the spin Hamiltonian, allowing for longitudinal magnetization exchange between coupled proton nuclei. Hence, the cross-peaks observed in the RFDR spectrum arise from proton magnetization exchange occurring via both the incoherent NOEs (as in NOESY) as well as via proton spin-diffusion process driven by the coherent recoupled ^1^H-^1^H dipolar interactions^33^,^34^. The XY4^1^_4_ phase cycling scheme was employed in the fp-RFDR experiments because it has been proven to be more efficient in providing better recovery of magnetization (i.e., less loss of magnetization usually caused by imperfections in π pulses and the cross-terms in the effective Hamiltonian) than other XY-based phase cycles, and hence it enables the application of more recoupling π pulses during the RFDR mixing period, enhancing the magnetization exchange efficiency^15^. It can be noticed from Fig. 4B that most cross-peak intensities in the RFDR spectrum are higher than their counterparts in the NOESY spectrum. For example, the aliphatic side chain protons have much higher cross-peak intensities in the RFDR spectrum as compared to those in the NOESY spectrum, which indicates that these side chains are associated with strong dipolar interactions with water and with the amide protons. Notably, it can be observed from Fig. 4 that no cross-peaks were observed for the structural hydroxyl group of the bone mineral, implying that these OH groups exhibits weak ^1^H-^1^H dipolar couplings with water and the bone organic matrix, which suggests that these groups are located within the mineral crystal lattice and not in close proximity to the hydrated organic-mineral surface layer in bone.

Since the NOEs and through-space dipolar couplings are both dependent on proton-proton distances (~1/*r*^6^ in the case of NOE and ~1/*r*^3^ for through-space dipolar couplings)^38^,^49^,^50^, then these present experiments can provide a valuable tool for proton-proton distance measurements that are quintessential for structure elucidation. In order to obtain a quantitative understanding of the magnetization exchange occurring through these two effects, it is important to analyze and interpret the time-dependent behavior of the cross-peak intensities as observed in the 2D RFDR spectra under ultrafast MAS conditions. This time-dependent behavior, represented by a build-up curve of the cross-peak intensity as a function of the RFDR mixing time, can be used to extract relaxation parameters. These RFDR build-up curves were obtained by fitting the experimental cross-peak intensities to a rate equation that has been explained in detail elsewhere^36^,^37^,^51^. Briefly, this differential rate equation describes the time evolution of the proton longitudinal magnetization (**M**), and is written as: *d***M**/*dt* = [**R** **+** **L**].**M**, where **R** and **L** are kinetic matrices^52^,^53^,^54^. The diagonal elements *R*_ii_ of matrix **R** are the auto-relaxation rates of spin *i*, while the off-diagonal elements *R*_ij_ describe the cross-relaxation rate between spins *i* and *j*. The elements *L*_ij_ of matrix **L** represent the magnetization exchange between spins *i* and *j* due to dipolar couplings and are given by *L*_ij_ = *D*_ij_/*r*^2^_ij_, where *D* is the diffusion constant and *r* is the distance between the two spins. The RFDR build-up curves were fitted to the experimental cross-peak intensities using a matrix diagonalization approach implemented in a home-made Matlab script that makes use of the above rate equation. These build-up curves have both exponential growth and exponential decay fit parameters, and three relaxation parameters were extracted from the best-fit build-up curves of the experimentally measured cross-peak intensities in the RFDR spectra: the observed relaxation rate (1/*T*_1_*), the build-up rate (*R*), and the peak intensity (*I*_0_). *T*_1_* includes the intrinsic spin-lattice relaxation *T*_1_ times of protons as well as imperfections in the recoupling π pulses applied during the RFDR mixing period.

**Figure 5:**
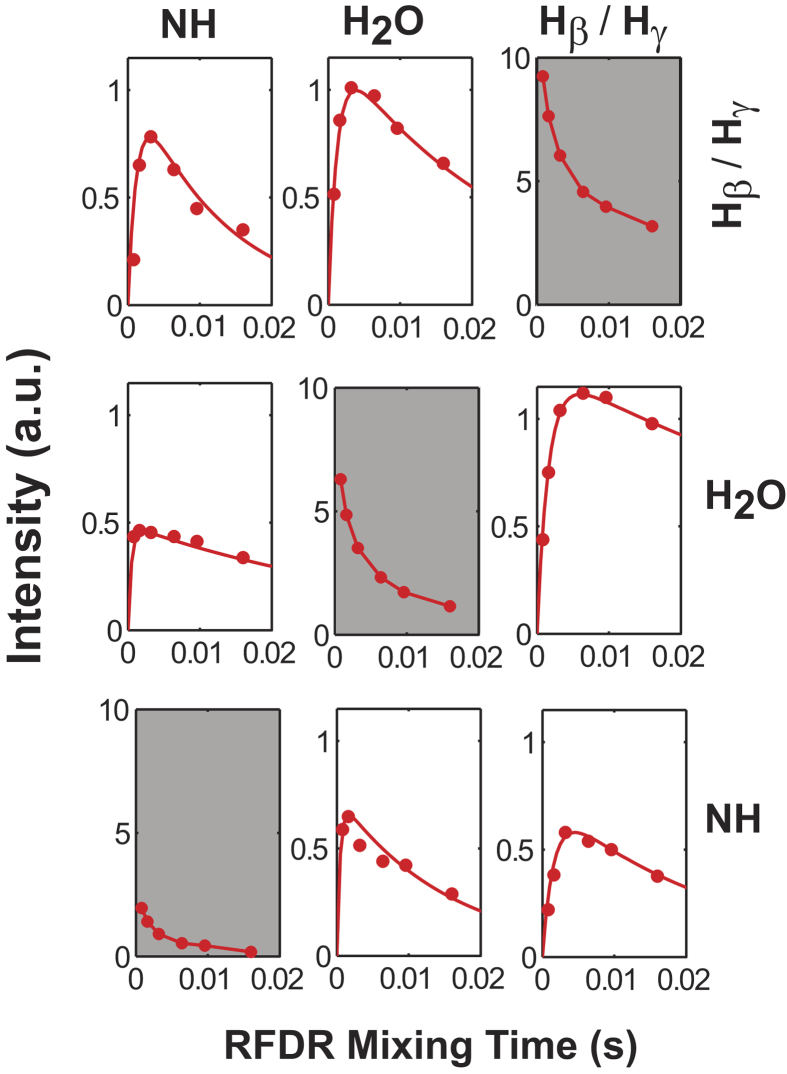
Experimentally measured build-up curves of the RFDR cross-peaks. Intensity scales were chosen arbitrarily to enable a direct comparison. The RFDR spectra were obtained for mixing times of 0.8, 1.6, 3.2, 6.4, 9.6, and 16 ms.

Figure 5 displays the evolution of diagonal and cross-peak intensities as a function of the RFDR mixing time. Experimentally determined peak intensities of all diagonal peaks and cross-peaks as a function of mixing time are shown as data points, while the line curves are the best fit to these intensities using the relaxation parameters listed in Table 1, as determined by the above-mentioned rate matrix equation. The calculated dynamic rates, and hence the simulated build-up curves, fit well with the measured peak intensity matrices obtained at 100 kHz MAS frequency over the 0.8–16 ms RFDR mixing time range. The difference in the decay behavior of the different diagonal peaks is apparent from the plots in Fig. 5, where the NH diagonal peak decays at a faster rate than the other diagonal peaks. Within 6.4 ms, the NH diagonal peak decays to 27% of its original value, whereas the water and H_β_/H_γ_ peaks decay to 37% and 49% of their initial values, respectively. This faster decay rate of the NH diagonal peak implies that a larger percentage of the magnetization is transferred to the relevant amide correlation cross-peaks, which reflects stronger ^1^H-^1^H dipolar couplings between the amide protons and those in their vicinity, suggesting a highly rigid structure of the backbone region of the collagen fibres in bone.

A careful inspection of the plots in Fig. 5 and the parameters in Table 1 reveals the modes of atomic-level interaction exhibited by water with the aliphatic and the amide regions of the organic matrix, where these interactions could explain the distribution as well as the role played by water in the mechanical function of bone. Figure 5 shows that the cross-peak intensity for the water-amide region reaches a maximum within a short mixing time of 1.6 ms, whereas the cross-peak intensity for the water-aliphatic region reaches a maximum after a longer time of of ~3.2–6.4 ms. The intensity of the water-amide cross-peak builds up at a much higher rate than that of the water-aliphatic cross-peak, which indicates that water exhibits a much stronger ^1^H-^1^H dipolar interaction with the amide region than with the side-chain region of collagen. The water-amide cross peak thus corresponds to water strongly interacting with the triple helix of tropocollagen molecules. As such, it would correspond to structural water incorporated as an integral component of the tropocollagen helical structure. The appearance of this cross-peak, and consequently the strength of the water-amide interaction, can be attributed to the fact that, at the atomic level, water molecules are involved in an inter-chain hydrogen bonding network that indirectly connects the carbonyl group of glycine in the (Gly-Xaa-Yaa) repeating unit of one collagen α-chain with the NH group of an amino acid residue in the adjacent chain^55^,^56^,^57^,^58^; these water bridges help to stabilize the triple-helical conformation of collagen in bone, and also contribute to the rigidity of the backbone region of collagen. On the other hand, the interaction of water with the polar side-chain residues of collagen gives rise to a cross-peak with a slow build-up rate compared to the water-amide peak, reflecting weaker ^1^H-^1^H dipolar couplings that are due to polar (van der Waals) interactions between mobile water and collagen side chains. These results also indicate the flexibility of collagen side chains, which is further reflected by a similar build-up behavior for the side chain-amide cross-peak. The results from the RFDR measurements under 100 kHz MAS provide a direct evidence for the interaction between water and the collagen molecules via the analysis of cross-peak intensities observed in the RDFR spectra, which might otherwise not be possible under slower MAS rates.

## Conclusion

To investigate the interaction between water and different domains of collagen at the highest possible atomistic resolution, we have reported the first application of ^1^H-detected multidimensional NMR measurements under ultrafast (up to 110 kHz) MAS on bone samples. The increase in ^1^H *T*_2_ transverse relaxation times with faster spinning is consistent with the homogeneous nature of the line broadenings observed in the ^1^H 1D NMR spectra. As such, narrower linewidths could be achieved with faster spinning, enabling considerable gain in both spectral resolution and sensitivity. Thus, the feasibility of proton detection under ultrafast MAS for structural elucidation by solid-state NMR spectroscopy using a very limited amount of bone samples is demonstrated. We have also demonstrated that 2D ^1^H/^1^H chemical shift correlation spectra for bone can be obtained using fp-RFDR pulse sequence under ultrafast MAS. Our calculated dynamic parameters, and hence our simulated cross-peak build-up curves, are in excellent agreement with the measured cross-peak intensities observed at 100 kHz MAS in the 2D ^1^H/^1^H spectra. Our results also infer that water exhibits different ^1^H−^1^H dipolar coupling networks with collagen backbone and side chain. These ^1^H−^1^H dipolar interactions suggest that the 2D ^1^H/^1^H chemical shift correlation experiments can be a potential tool for monitoring mechanical loading and disease-associated structural and dynamic changes in bone and other collagen-based systems. More generally, ultrafast MAS frequencies of 100 kHz and higher can yield remarkable resolution enhancements in multidimensional homo- and hetero-nuclear NMR measurements on solid systems of chemical, biological, pharmaceutical and material significance. Furthermore, the resolution and sensitivity enhancements afforded by ultrafast MAS in ^1^H-detected NMR experiments provide new opportunities for the investigation of insoluble non-crystalline solids that can neither be studied by solution-state NMR spectroscopy nor by other diffraction techniques.

## Methods

Powdered bovine cortical bone samples were harvested from bovine femora collected at a local slaughterhouse from freshly slaughtered animals (2–4 years old). All animal procedures were carried out in accordance with the relevant guidelines and standards of the University of Michigan, and were approved by the University Committee on Use and Care of Animals (UCUCA). After cleaning off soft tissues, slices of cortical bone were dissected from middle diaphyseal femoral sections and cut into rectangular specimens with a band saw under continuous irrigation with calcium-buffered saline to prevent overheating of bone and keep Ca balance. Rectangular specimens were then ground into powder using a cryogenic mill under liquid nitrogen. Finally, the powdered samples were soaked with calcium–buffered PBS (Ca-PBS) solution and stored at −20 °C prior to NMR measurements.

All ^1^H-detected solid-state NMR experiments were conducted at 14.1 T on a 600-MHz JEOL solid-state NMR spectrometer (JNM-ECA600II) operating at a ^1^H Larmor frequency of 600.17 MHz, and equipped with a 0.75-mm double-resonance ultrafast MAS probe (JEOL RESONANCE Inc., Tokyo, Japan). All measurements were carried out at 25 °C using about 0.3 mg of bone sample packed into 0.75-mm MAS zirconia rotor (290 nL volume). One-dimensional (1D) ^1^H MAS spectra were recorded at multiple MAS frequencies (that ranged from 20 to 110 kHz) by co-adding 4 transients using a rotor-synchronized Hahn spin-echo (90°-τ_r_-180°-τ_r_) pulse sequence with a π/2 ^1^H pulse duration of 0.72 μs and a recycle delay of 3 s. The transverse relaxation times (*T*_2_) were measured at each spinning frequency using the Hahn spin-echo sequence, where the echo delay was incremented. The *T*_2_ values were obtained by fitting the measured signal integral intensities to a mono-exponential decay function. The two-dimensional (2D) ^1^H/^1^H chemical shift correlation spectra were collected under 100 kHz MAS using a finite-pulse RFDR pulse sequence with the 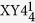 phase cycling scheme during the mixing time to recouple zero quantum ^1^H-^1^H dipolar interactions. These spectra were acquired with an acquisition time of 5.12 ms, 512 *t*_2_ complex points, a 0.72 μs 90° ^1^H excitation pulse, a 3 s recycle delay, 32 *t*_1_ increments and 36 transients per increment. A series of six mixing times between 0.8 ms and 16 ms was used to construct build-up curves for the cross-peaks observed in the RFDR spectra. Under the same experimental conditions, a 2D NOESY spectrum was also collected on the same bone samples under 100 kHz MAS using a mixing time of 32 ms. All 2D spectra were processed with a squared sine bell apodization (90° shift) and zero filling to 1024 points in each dimension.

## Additional Information

**How to cite this article**: Mroue, K. H. *et al.* Proton-Detected Solid-State NMR Spectroscopy of Bone with Ultrafast Magic Angle Spinning. *Sci. Rep.* **5**, 11991; doi: 10.1038/srep11991 (2015).

## Figures and Tables

**Table 1:** The best-fitting parameters for the build-up curves extracted from the fp-RFDR based 2D ^1^H/^1^H chemical shift correlation spectra.

cross-peak	1/T_1_^*^(s^−1^)	R (s^−1^)	I_0_
H_β_/H_γ_-H_2_O	15	310	2.5
H_β_/H_γ_-NH	42	302	1.5
H_2_O-NH	64	1050	1.5
H_2_O- H_β_/H_γ_	40.2	390	2.45
NH- H_2_O	25	1040	0.98
NH- H_β_/H_γ_	80	380	2.2
